# You are How You Eat: Foraging Behavior as a Potential Novel Marker of Rat Affective State

**DOI:** 10.1007/s42761-024-00242-4

**Published:** 2024-06-26

**Authors:** Vikki Neville, Emily Finnegan, Elizabeth S. Paul, Molly Davidson, Peter Dayan, Michael Mendl

**Affiliations:** 1 Bristol Veterinary School, University of Bristol, Langford, UK; 2 Max Planck Institute for Biological Cybernetics & University of Tübingen, Tübingen, Germany

**Keywords:** Affective state, Behavior, Foraging, Rat, Rodent

## Abstract

Effective and safe foraging requires animals to behave according to the expectations they have about the rewards, threats, and costs in their environment. Since these factors are thought to be reflected in the animals’ affective states, we can use foraging behavior as a window into those states. In this study, rats completed a foraging task in which they had repeatedly to decide whether to continue to harvest a food source despite increasing time costs, or to forgo food to switch to a different food source. Rats completed this task across two experiments using manipulations designed to induce both positive and negative, and shorter- and longer- term changes in affective state: removal and return of enrichment (Experiment 1), implementation and reversal of an unpredictable housing treatment (Experiment 1), and delivery of rewards (tickling or sucrose) and punishers (air-puff or back-handling) immediately prior to testing (Experiment 2). In Experiment 1, rats completed fewer trials and were more prone to switching between troughs when housed in standard, compared to enriched, housing conditions. In Experiment 2, rats completed more trials following pre-test tickling compared to pre-test sucrose delivery. However, we also found that they were prone to disengaging from the task, suggesting they were really choosing between three options: ‘harvest’, ‘switch’, or ‘not work’. This limits the straightforward interpretation of the results. At present, foraging behavior within the context of this task cannot reliably be used as an indicator of an affective state in animals.

**Supplementary Information:**

The online version contains supplementary material available at 10.1007/s42761-024-00242-4.

To maximise fitness, in broad terms the ability to survive and reproduce, individuals face an intricate balancing act between minimising energetic expenditure, maximising reward intake (e.g. food), and avoiding punishers (e.g. predation). Knowing how best to balance these ultimately depends on the sort of environment an individual inhabits. Greater effort should be directed to vigilance behaviors in high predation environments, and to obtaining rewards in periods when they are particularly and cheaply abundant. Accordingly, three of the most critical components of an individual’s expectations about their environment are opportunities for reward, the presence of potential threats, and the existence of costs.

An influential conception is that the expectations an individual holds about their environment are reflected in, or perhaps comprise, their affective state (Mendl & Paul, 2020; Mendl et al., 2010), and it appears that the critical components of the environmental expectations described are coupled to states such as anxiety and depression. For example, an increased expectation of punishers, and an urgency to avoid, are thought to be linked to anxiety (Bishop & Gagne, 2018; Fung et al., 2019; Mendl & Paul, 2020; Mendl et al., 2010). Equally, a decrease in the expectation of rewards or the effort individuals are willing to expend to obtain them is thought to be coupled to depression (Bishop & Gagne, 2018; Horne et al., 2011; Mendl et al., 2010; Mendl & Paul, 2020). This means that ways of measuring such expectations will provide a window onto an individual’s affective state. Such a window would be of particular value for non-human animals, given the vital role they play in studies of the aetiology and treatment of mood disorders and because refined measures of their welfare (which can be defined in terms of affect) are required (Neville, Mendl et al., 2023).

Foraging tasks, in which animals have to engage with different locations to obtain food, are of particular value as measurement methods since all three components of environmental expectation can influence the willingness to overcome the cost and danger of switching to new locations (Shettleworth & Plowright, 1989; Wilson et al., 2021; Addicott et al., 2017). A common, simplified, example is a so-called bandit task. Here, the locations might be different levers or nose pokes; and the choice of engagement involves pressing or poking. Each location is a ‘bandit’ (as in a casino) in that engaging with it leads to the delivery of a valued outcome (e.g. food) with a unique probability that is not known to the animal a priori (Shettleworth & Plowright, 1989; Costa et al., 2019; Cinotti et al., 2019). These tasks afford the experimenter a high degree of control over the environment, and so what the animal should come to expect: it is possible to manipulate the amount of food available in different locations, variation in food availability, as well as the distance between and potential for punishers in different locations. While these tasks have been widely used to study the neurobiology of decision-making in non-human animals (Costa et al., 2019; Cinotti et al., 2019; Wilson et al., 2021; Addicott et al., 2017), little attention has been paid to how behavior in these tasks might relate to their affective states.

This lack of attention is perhaps surprising. Firstly, animals must eat and drink to survive, making foraging a ubiquitously critical concern across species. Secondly, there is increasing emphasis on the role of individual differences in studies of animal affect and welfare, both in terms of its influence on a particular measure of affect and its influence on manipulations designed to alter affect (Neville, Lind et al., 2023; Richter & Hintze, 2019; Brooks et al., 2024). Given the pervasiveness of foraging behavior, it is an excellent candidate behavior for studies in this area. And finally, because empirical studies with humans have indicated that an individual’s affective state may indeed be linked to their behavior in such tasks. Such studies have reported increased persistence (e.g. staying at the same foraging patch) in those with poorer mood states (van Dooren et al., 2021; Aberg et al., 2022; Blanco et al., 2013; Harlé et al., 2017), although there are also opposing results (Lenow et al., 2017; Strauss et al., 2011). The subtlety and opportunity of foraging tasks as sensitive measuring instruments is evident in the fact that both these possibilities are consistent with different aspects of the theoretical account of the links between mood disorders and behavior. A belief that the wider environment is poor or increased estimates of the effort costs, which have been associated with poorer moods (Lenow et al., 2017; Horne et al., 2011), could lead to increased persistence (Lenow et al., 2017; Addicott et al., 2017). While, high levels of uncertainty about the state of the environment or a reduced valuation of rewards, which have equally been associated with poorer moods (Clark et al., 2018; American Psychiatric Association, 2013), could lead to a reduction in how willing an individual is to persist in foraging at one source (Harlé et al., 2017; Addicott et al., 2017).

The aim of this study was, therefore, to assess whether foraging behavior in an operant task might vary with the affective state in rodents. To achieve this, we trained and tested rats on a foraging task translated from a study with human subjects described by Lenow et al. that had been framed in the context of foraging decisions. In this, rats had to choose between staying with a food source with increasing harvest costs (increased wait time to obtain food) or moving to a new food source. We used a range of affect manipulations: both short- and long-term, and both positively- and negatively-valenced. In Experiment 1, rats experienced changes in housing conditions commonly used to alter affect (either enrichment removal/addition or unpredictable housing addition/removal), and in Experiment 2, given that reward and punisher experience is thought to be a key determinant of affect according to theoretical conceptualisations (Mendl et al., 2010; Rolls et al., 2005; Mendl & Paul, 2020), rats experienced delivery of known rewards (tickling; LaFollette et al., 2017, or sucrose; Smith & Berridge, 2005) or punishers (air-puffs; Moriarty et al., 2012, or back-handling — grasping a rat around their thorax behind their forelegs and lifting them up; Lorenzini et al., 1990) immediately prior to testing. We predicted that foraging behavior would be influenced by the affect manipulations, but were agnostic to the direction of any effects, given the conflicting results in the human literature. We had hoped to gain a more complete understanding of the links between affect and behavior using computational modelling within a marginal value theorem framework (Charnov, 1976), following Lenow et al. However, ‘switch’ decisions became conflated with periods of disengagement from the task — which we had not anticipated given the existing literature on bandit tasks — meaning that this was only partly possible.

## Method

### Ethics Statement

This study was conducted at the University of Bristol and received ethical approval (UK Home Office Project License no: P2556FBFE). Animal use and care were in accordance with the UK Animals (Scientific Procedures) Act 1986 (ASPA) and the UK Home Office code of practice for the housing and care of animals bred, supplied, or used for scientific purposes. All animals were regularly weighed and monitored throughout the study for any health issues. At the end of the study, rats were re-used in a separate study before eventually being rehomed as pets. We adhered to the ARRIVE guidelines for reporting animal research when preparing this article (Percie du Sert et al., 2020).

### Subjects and Husbandry

Subjects were 40 male Lister-hooded rats (Envigo, UK) who weighed 100–125 g on arrival. Only male animals were used due to restrictions on space, and because the majority of the research that informed our choice of affect manipulations had been conducted with male rats (Neville, Lind et al., 2023; LaFollette et al., 2017). All 40 of these rats were used in Experiment 1 and only 20 were used in Experiment 2. A power analysis was conducted to calculate the number of animals required to detect treatment differences in decision-making using binomial generalised linear mixed models, based on effect sizes reported by Neville et al. (2020). These effect sizes were derived from the beta coefficients for the effect of short-term rewards (air-puff pre-treatment vs. sucrose pre-treatment) in a binomial generalised mixed model of differences in decision-making (‘stay in trough’/‘leave trough’) on an automated judgement bias task. The experimental unit was the animal. The analysis suggested that approximately eighteen animals in each treatment group would achieve a power of .9. To account for the possibility that animals failed to learn the task, did not perform sufficient trials during testing, or became ill, an additional two animals were included per treatment group.

Animals were pair-housed across two colony rooms, with the allocation of rats to a cage or colony room conducted by animal care staff. Rats were kept under a 12-h reverse light cycle, with lights off at 7am and on at 7pm. Rats were housed in a standard rat cage (Techniplast 1500U Eurostandard) that had been extended using a custom-made cage topper to provide a mezzanine floor and an area in the cage where the animals could rear fully (entire cage measuring 480(L) $\times$ 375(W) $\times$ 420(H) mm). Cages contained aspen bedding, nesting material, and the following enrichment items: a red suspended perspex box, a cardboard tube, a cardboard nest box, and an aspen block (see Supplementary Material for photographs of the caging). Rats had ad libitum access to food (LabDiet) and water. In preparation for the tickling affect manipulation, rats were tickled three times per week (the minimum dosage recommended to familiarise rats with tickling, LaFollette et al., 2018) until the start of testing when they were only tickled once per week to maintain familiarity with tickling. During these tickling sessions, ultrasonic vocalisations were monitored in real time using a bat detector (Echo Meter Touch Bat Detector 2, Wildlife Acoustics) connected to an iPad. These data were not saved. This was conducted on a non-training day to avoid any potential influence of the tickling on behavior on the task. All rats were handled by scooping for day-to-day handling (e.g. when being moved in/out of the homecage): placing the hand underneath the abdomen and lifting with a loose grip around the rat’s body, and allowing the rat to rest in the hand or on the forearm.

### Foraging Task Training

Prior to training, rats were habituated to shuttle boxes (measuring 508(L) $\times$ 254(W) $\times$ 305(H) mm). This involved putting the rats with their cagemate in the shuttle box with neither of the troughs located on the two end walls of the shuttle box accessible. Instead, a bowl of sucrose pellets (45 mg per pellet; Bioserv Rodent Dustless Precision Pellets) was available for 15 min. This was repeated the following day with only one rat per shuttle box. Training on the task comprised three stages. The aim of the first stage was to train rats to associate visits to the shuttle box troughs with sucrose pellets. This stage consisted of four training sessions each of 15 min duration in which rats could only access one side of the shuttle box (with a wall placed centrally to prevent access to the alternate side): two conducted with rats placed in one side of the shuttle box, and two conducted with rats in the other side. One rat in each cage would have their first training session in the left compartment, and the other rat would have their first test session in the right compartment. This side would switch for each session. In these sessions, one sucrose pellet was delivered 20ms after the rat’s snout entered the trough. The aim of the second training stage was to introduce the rat to the both troughs being available to visit. In the second training stage, which comprised two sessions, the training sessions were increased to 30 min, and both sides of the shuttle box were accessible with troughs on each side delivering one sucrose pellet 20ms after the rat’s snout entered the trough. The aim of the final training stage was to introduce the rats to the increasing time delay required to obtain sucrose for consecutive visits to the same trough. These training sessions were identical to the test sessions (outlined below) and rats completed one of these sessions before progressing to testing.

**Table 1:** Summary of the affect manipulations used in Experiments 1 and 2

	Experiment 1: Long-term (3 weeks each)	Experiment 2: Short-term (5 min each)	References
Negative affective induction	Unpredictable housing enrichment removal	15 $\times$ air-puffs 15 $\times$ back handling events	Brydges et al. (2011); Burman et al. (2008); Engelmann et al. (1996); Lorenzini et al. (1990); Neville et al. (2020); Neville, Lind et al. (2023); Willner (2017)
Positive affect induction	Unpredictable housing reversal enrichment return	15 $\times$ sucrose pellets 15 $\times$ tickling events	Brydges et al. (2011); LaFollette et al. (2017); Neville et al. (2020; Neville, Lind et al. 2023); Sclafani and Ackroff (2003)

### Foraging Task Testing

Test sessions had a duration of 30 min. This duration was selected as it has previously worked well in studies that we have conducted with rats: providing a balance between collecting sufficient data, without issues of satiation (Jones et al., 2018; Neville et al., 2020). Our previous research has shown that rats will consume 50 sucrose pellets in an average of 5 min, and an average of well over 200 pellets in an hour, when they are freely provided, and self-initiate trials at a rate of 4–5 trials/min in test sessions of 30-min duration (Parker, 2008; Jones et al., 2018). On the initial visit to each trough, and on visits where the rat had previously been at the other trough (i.e., a trough was reset as soon as the other trough was visited), rats were required to keep their snout in the trough for a duration randomly selected from between 0 and 100 ms before a pellet was delivered into the trough. This duration increased on consecutive visits to the same trough by a value randomly selected between 100 and 200 ms. If the rat did not stay in the trough for the required time then no sucrose pellet was delivered. These timings were selected as previous studies have demonstrated that rats will readily learn to keep their snout in a trough for at least 2 s to obtain a sucrose pellet when only one trough is available (Neville et al., 2020; Jones et al., 2018). Rats therefore had to make a choice between staying at the same trough with an increasing ‘harvest time’ for sucrose (‘harvest’) or forgoing sucrose to move to the other trough to obtain sucrose at that location (‘switch’). Both troughs operated on the exact same principles and time delay sequences. The collection of data on this task (i.e., time of entry to and exit from each trough) was automated, ensuring that that there was no observer bias during testing (data effectively being collected blind).

### Affect Manipulations

In Experiment 1, there were 7 weekly test sessions: one baseline test session, followed by three test sessions conducted while the animals underwent the long-term affect manipulation, followed by an additional three test sessions after rats had been returned to baseline conditions. The affect manipulations, unpredictable housing or enrichment removal, were used as previous work has indicated that these treatments generate poorer environments that lead to negative affective states — with some evidence that their reversal also generates a relatively positive affective state, see Table 1 (Neville, Lind et al., 2023; Willner, 2017; Brydges et al., 2011; Burman et al., 2008; Hill et al., 2012; Paolo et al., 1994).

Half the rats underwent an unpredictable housing manipulation, which involved use of a chronic mild stress protocol (Willner, 2017), for a period of 3 weeks. This involved (max duration (h) and max frequency/wk in parentheses): bedding dampened by 100 ml water (4 h, 1/wk); light cycle reversed (3 h, 3/wk); cage tilted (1 h, 1/wk); white noise from radio (1 h, 1/wk); cage topper removed (4 h; 1/wk). Events could occur at any time during the dark phase of the light cycle, excluding a period 2 h before or after testing with no more than two events on any given day that did not overlap. The remaining rats were moved from enriched to standard housing (the cardboard nestbox and cage extending topper, which included the suspended red box, were removed) in the same period. As the unpredictable housing treatment involved colony room-wide manipulations, assignment to a treatment was based on colony room allocation (i.e., all rats in colony room A underwent unpredictable housing, and all rats in colony room B underwent enrichment removal). Analysis of data from the first baseline session confirmed that there were no significant differences in foraging behavior between the colony rooms prior to the start of the affect manipulations (Number of trials initiated: LRT = 0.007, *p* = .934; Proportion of ‘switch’ trials: LRT = 1.440, *p* = .230). All rats were returned to their baseline conditions after these manipulations and studied for a further three test sessions thus allowing us to evaluate the effects of the induction of relatively negative (treatment) and positive (return to baseline) states using both addition/removal of threats (unpredictable housing) and loss/gain of resources (enrichment).

In Experiment 2, each rat had 2 weekly test sessions for 5 weeks: one wash-out test session followed by an affect manipulation (to reduce the effect that the previous affect manipulation might have on the next test session, and enhance the subsequent affect manipulation), and subsequently, a test session in which an affect manipulation preceded testing. The data from the wash-out test sessions were not analysed. The specific treatments here were selected as they would be anticipated to alter affect according to theoretical conceptualisations of this construct. Positive and negative affect have been operationally defined as states elicited by rewards and punishers/threats, where rewards are appetitive stimuli — things an animal will approach or work to obtain and that aid the individual’s survival, and punishers/threats are aversive stimuli — things an animal will avoid or work to avoid and that may be detrimental to survival (Rolls et al., 2005; Mendl & Paul, 2020; Paul & Mendl, 2018). Similarly, motivational theories of basic or discrete emotions also anchor them in behavior. For example, states of ‘fear’ or ‘basic fear’ are viewed as occurring in response to dangerous (punishing) situations and motivating adaptive responses (e.g., freezing, fleeing) that help the animal to avoid damage (Scarantino, 2014, 2018). Accordingly, the treatments in Experiment 2 comprised repeated exposure to rewarding (found to be appetitive as they elicit an approach response, and so by definition induce positive affect) or punishing/threatening (found to be aversive as they elicit an avoidance response, and so by definition induce negative affect) events. Specifically, these treatments were: delivery of 15 sucrose pellets at random intervals within 5 min, delivery of 15 air-puffs at random intervals within 5 min, 5 min in cage with no experimenter intervention (control), 5 min of tickling, 5 min of back-handling, see Table 1. Sucrose is widely used as a food reward for rodents — they readily approach and consume sucrose, and conversely, rats will avoid air-puffs suggesting that air-puffs arepunishing (Neville et al., 2020; Engelmann et al., 1996; Sclafani & Ackroff, 2003). Tickling is considered to mimic playful social interactions in rats and there is evidence that rats will work (press a lever) to be tickled, while back handling mimics a predatory attack and there is evidence that it is aversive to rats (Burgdorf & Panksepp, 2001; LaFollette et al., 2017; Lorenzini et al., 1990).

Cumulative experience of rewarding and punishing events generates fluctuations in baseline affect or mood-like states (e.g., Turner and Lloyd, 1995; Kessler, 1997; Mendl & Paul, 2020) which can thus provide information about recent and ongoing environmental levels of reward and punishers and hence allow individuals to make decisions that maximise reward (e.g., food) and successfully avoid punishers (e.g., predation), whilst balancing energetic expenditure in a manner that is most appropriate for their environment (Mendl et al., 2010; Mendl & Paul, 2020). Quantifying how an individual acts to achieve this balance is what our task is attempting to measure. We thus consider that the affective state of the animal entering the test session (as induced by the preceding shorter or longer-term manipulations) will impact behavior throughout the test session, even if the test session itself subsequently altered affect through the use of sucrose pellets (Neville et al., 2021). Previous research has shown that such treatments are effective in similar tasks (Neville et al., 2020).

Each manipulation was conducted in the rats’ home-cage that had been moved to the experimental room and both rats in the cage received the same manipulation simultaneously. To deliver the sucrose pellets, the experimenter held a pot of sucrose pellets in front of each rat. To administer the air-puff, the experimenter aimed the air nozzle at the rat and then activated a solenoid valve connected to a medical air cylinder via a hand control switch. The resulting air-puff had a pressure of 50psi. The experimenter released the hand control switch to stop the air-puff as soon as the rat moved away. The back handling involved picking each rat up in turn with one hand by grasping the rat around their shoulders. Tickling followed the Panksepp method (Cloutier et al., 2018) in which dorsal contact was made before flipping and pinning the rat, this was repeated three times with a gap between each tickling bout while the rat’s cage-mate was tickled. The order of treatments was pseudorandomised for each cage such that exactly two cages received one of the five treatments each week with rats only receiving each treatment on one of the five test weeks.

### Data Analysis

All 40 rats completed training and testing, and no data were excluded from the analyses. The analyses for Experiment 1 were conducted on data from 40 rats (20 per treatment group), each completing seven test sessions with a total of 32,075 trials. Analyses for Experiment 2, were conducted on data from 20 rats (all undergoing each treatment), each completing 5 test sessions (one for each treatment including the control treatment) with a total of 25,662 trials. Analyses were conducted in R, by a researcher who was not blinded to the treatment groups. The assumptions of normality and homoscedasticity of residuals were met.

The foraging task generates two key outcome measures: the decisions made on each trial (i.e., ‘harvest’ or ‘switch’), and the total number of trials completed during each test session (given that rats are free to initiate as many trials as they like within the 30 min test session). Here, we define a trial as being initiated when a rat leaves a trough and ending when they revisit a trough, either the same trough of the alternative trough. These measures are inevitably related because longer delays between trial initiations mean that a rat can complete fewer trials. We assessed the extent of the dependence between these measures by fitting a general linear mixed model (GLMM) with subject as a random effect to the number of trials completed or the proportion of ‘switch responses’ made within the same test session. GLMMs that additionally included a top-level random effect of cage to account for rats being pair-housed either led to singularity issues or gave results that were identical to different models that did not account for cage, indicating that it was not possible to include this factor. A likelihood ratio test was used to assess significance. A simple correlation metric was also obtained using Spearman’s $\rho$ for these data across all rats and all sessions.

To assess if and how decision-making in the foraging task varied according to the affect manipulations, a binomial generalised linear mixed model (binomial GLMM) was fitted to the decision made on each trial (0=‘harvest’,1=‘switch’) with subject nested within test session as random effect. Likelihood ratio tests were used to assess the significance of fixed effects.

For Experiment 1, we first conducted a broader analysis to examine the question of whether the affect manipulations altered behavior. For this, we included trial number, session order (i.e., 1–7), environment (baseline or affect manipulation), manipulation type (unpredictable housing or enrichment removal), as well as the interaction between environment and manipulation type as fixed effects in the binomial GLMM. Where the interaction was significant, binomial GLMMs were also fitted to subsetted data (unpredictable housing or enrichment removal only/each test session only) to examine the The specific effect that each manipulation type had on decision-making. To examine the time course of the affect manipulations, where significant in the broader analysis, we fitted binomial GLMMs to the data that included just session order (as a factor), trial number, manipulation type as fixed effects and then conducted pairwise comparisons between sessions using Tukey’s honest significant difference test (which returns *p*-values adjusted for multiple testing). These analyses were also conducted separately for each manipulation type where the broader analysis had identified a significant interaction between environment and manipulation type. We only report where these pairwise comparisons are significant but all results are given in Supplementary Material.

In Experiment 2, treatment (control, tickling, sucrose, air-puff, or back-handling) was also included as a fixed effect as well as trial number and session order, and pairwise comparisons between treatments were made using Tukey’s honest significant difference test (which returns *p*-values adjusted for multiple testing). To assess if and how the number of trials completed in each test session varied according to the affect manipulations, the above analyses were repeated with the following changes: (1) a GLMM was instead fitted to these data with subject as a random effect, (2) trial number was not included as a fixed factor.

## Results

### Experiment 1

**Fig. 1:**
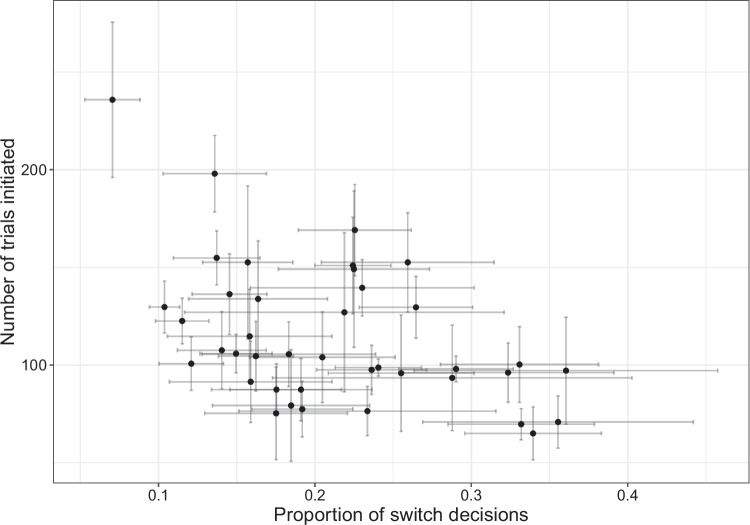
Scatterplot of the number of trials initiated versus the proportion of switch decisions. Each point represents the mean value for an individual rat, with the error bars showing the standard error

Rats completed an average of 115 ± 5.7 trials across all test sessions in Experiment 1. They made the ‘harvest’ decision on an average of 79 ± 1.2% of trials, making a ‘switch’ decision after an average of 5.4 ± 0.35 consecutive trials at the same trough. It took rats an average of 33 ± 1.5 s to initiate a trial at the alternative trough following the end of a trial at the current trough, compared to an average inter-trial interval of 11 ± 0.61 s when rats made a ‘harvest’ decision. The number of trials initiated was significantly associated with the proportion of switch decisions for the same rat on the same test session (Fig. 1: $$\rho $$ = $$-$$.44, $$\beta \pm $$*SE* = $$-$$164±24, LRT = 43, *p*$$ < .001$$).

**Fig. 2:**
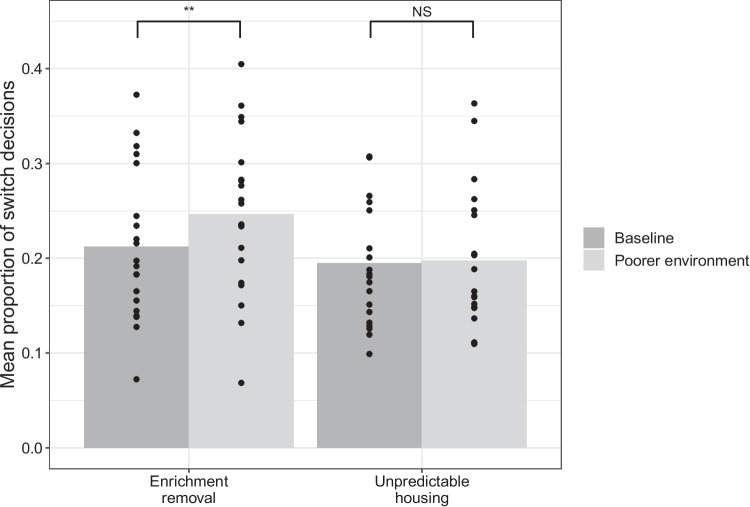
Mean proportion of switch decisions in the baseline and poorer environmental conditions for both the enrichment removal and unpredictable housing groups. Points represent the mean data for each individual rat. The asterisks denote the significance level: NS indicates non-significant, *indicates *p*$<.05$, **indicates *p*$<.01$, ***indicates p$<.001$

**Fig. 3:**
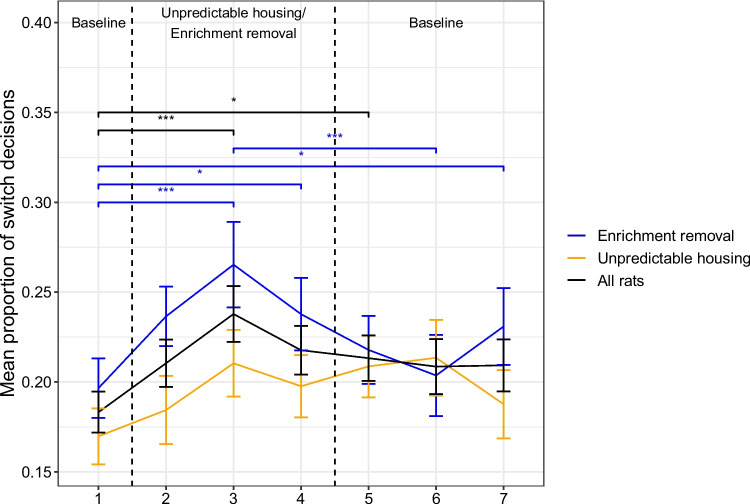
Mean proportion of switch decisions in each test week for each manipulation. Significant differences between different sessions are indicated by the horizontal lines with the significance level denoted by the asterisks; *indicates *p*$<.05$, **indicates *p*$<.01$, ***indicates *p*$<.001$. Error bars represent one standard error

**Fig. 4:**
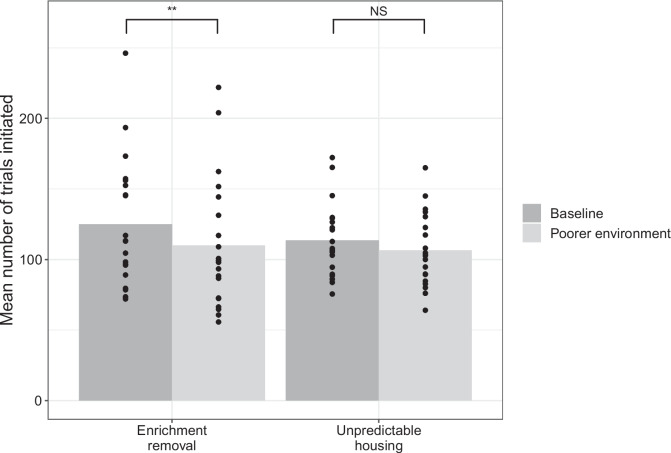
Mean number of trials in each state in the baseline and worse environmental conditions for both the enrichment removal and unpredictable housing groups. Mean Error bars represent one standard error. The asterisks denote the significance level: NS indicates non-significant, *indicates *p*$<.05$, **indicates *p*$<.01$, ***indicates *p*$<.001$

**Fig. 5:**
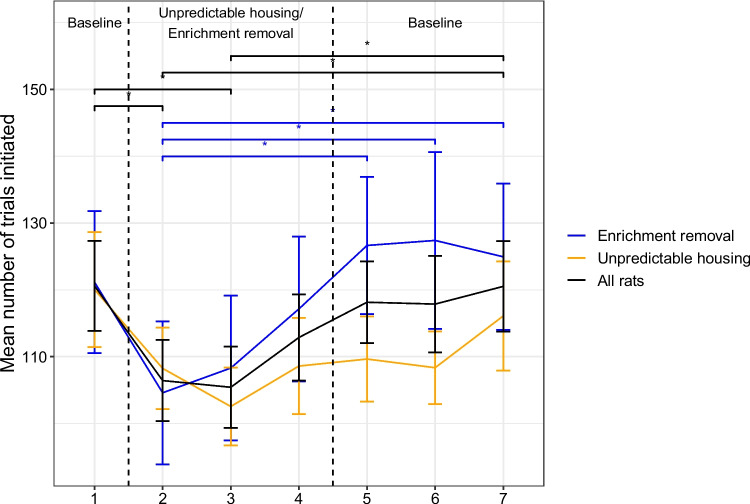
Mean number of trials initiated in each test week for for each manipulation type. Significant differences between different sessions are indicated by the horizontal lines with the significance level denoted by the asterisks; *indicates *p*$<.05$, **indicates *p*$<.01$, ***indicates *p*$<.001$. Error bars represent one standard error. Note that significant differences between sessions are presented for consistency with the plot of the ‘switch’ decision data (Fig. 2), but are not reported in the text as the interaction between manipulation type (enrichment removal vs. unpredictable housing) and environmental condition (baseline vs. poor) is not significant

**Fig. 6:**
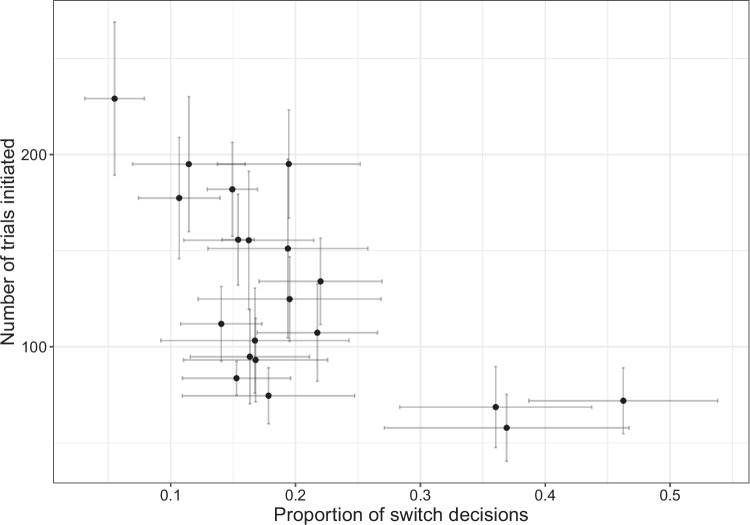
Scatterplot of the number of trials initiated versus the proportion of switch decisions for all test sessions in Experiment 2. Each point represents the mean value for an individual rat, with the error bars showing the standard error

Rats were more likely to ‘switch’ when they lived in poorer environments (Fig. 2: $\beta \pm$*SE* = 0.13 ± 0.039, LRT = 11, *p*$<.001$). More specifically, the post-hoc analyses revealed that rats were more likely to switch in test sessions 3 (*z* = 4.6, *p* $<.001$) and 5 (*z* = 3.1, *p* = .031) compared to test session 1 (Fig. 3). They were also less likely to ‘switch’ as the test session progressed ($\beta \pm$*SE* = $-$0.31 ± 0.019, LRT = 261, *p*$<.001$) and more likely to ‘switch’ after they had completed a greater number of test sessions ($\beta \pm$*SE* = 0.029 ± 0.010, LRT = 8.8, *p* = .003). Manipulation type was not a significant predictor of the decision made (LRT = 1.9, *p* = .17). However, the interaction between environment and manipulation type was significant (LRT = 5.8, *p* = .016): there was a significant effect of environment when the enrichment removal manipulation was used ($\beta \pm$SE0.21±0.047, LRT = 19, *p*$<.009$) but not when unpredictable housing was used (LRT = 0.48, *p* = .63), although there was no significant difference between the enrichment removal and unpredictable housing treatment at any timepoint (W (Week) 1: LRT = 1.1, *p* = .44, W2: LRT = 3.4, *p* = .14, W3: LRT = 3.5, *p* = .14, W4: LRT = 2.4, *p* = .22, W5: LRT = 0.15, *p* = .71, W6: LRT = 0.14, *p* = .71, W7:LRT = 3.5 *p* = .14). Further post-hoc analyses indicated that rats who underwent the enrichment removal manipulation were more likely to switch in sessions 3 (*z* = 5.0, *p*$< .001$), 4 (*z* = 3.1, *p* = .031), and 7 (*z* = 3.3,*p* = 0.019) compared to session 1, and session 3 compared to session 6 (*z* =$-$4.6, *p*$< .001$). There was no significant difference between any of the test sessions in the unpredictable housing group.

Rats also completed fewer trials when they lived in poorer environments ($\beta \pm$*SE* = $-$11±2.9, LRT = 14, *p*$<.001$; Fig. 4). In particular, rats completed fewer trials on sessions 2 (z=$-$3.0, *p* = .42) and 3 (*z* = $-$3.2, *p* = .02) compared to session 1, and also compared to to session 7 (S2 vs. S7: *z* = 3.0, *p* = .044; S3 vs S7: *z* = 3.2, *p* = .02); Fig. 5. Unlike the ‘switch’ behavior this did not depend on the manipulation type (Manipulation type*Environment: LRT = 2.4, *p* = .12). Manipulation type was not a significant predictor of the number of trials completed as a main effect (LRT = 0.52, *p* = .47) nor was number of test sessions (LRT = 0.02, 0.89).

### Experiment 2

In Experiment 2, rats completed an average of 128 ± 11 trials across all test sessions. They made the ‘harvest’ decision on an average of 80 ± 1.5% of trials, making a ‘switch’ decision after an average of consecutive 12 ± 1.6 trials at the same trough. Rats took an average of 31 ± 2.5 s to initiate a trial at the alternative trough following the end of a trial at the current trough (‘switch’ decision), and 12 ± 1.5 s to initiate a trial at the same trough (‘harvest’ decision). The number of trials initiated was significantly associated with the proportion of switch decisions for the same rat on the same test session (Fig. 6: $\rho$ = $-$.63, $\beta \pm$*SE* = $-$256±29, LRT = 68, *p*$<.001$).

Treatment was not a significant predictor of the decision to ‘switch’ or ‘harvest’ (LRT = 3.5, *p* = .48). The likelihood of rats making a switch response reduced as a test session progressed ($\beta \pm$*SE* = $-$0.22±0.02, LRT = 118, *p*$<.001$), and as rats completed more test sessions ($\beta \pm$*SE* = $-$0.044 ± 0.007, LRT = 32, *p*$<.001$). None of the pairwise comparisons between the control test sessions and other treatments were significant (Tables 2 and 3).

**Table 2:** The mean and standard deviation (*SD*) of the proportion of switch decisions and number of trials completed across the different treatments

Variable	Treatment	Mean	*SD*
Proportion switch	Air-puff	0.19	0.020
	Back-handling	0.20	0.017
	Control	0.19	0.016
	Sucrose	0.21	0.015
	Tickling	0.19	0.018
Number of trials	Air-puff	131	10
	Back-handling	129	7.9
	Control	128	7.6
	Sucrose	119	8.1
	Tickling	135	9.3

**Table 3:** Results of the pairwise comparisons of the proportion of switch decisions between the control and treatment sessions

Pairwise comparison	*z*-value	*p*-value
Back-handling vs. Air-puff	1.2	.78
Control vs. Air-puff	0.52	.99
Sucrose vs. Air-puff	1.6	.48
Tickling vs. Air-puff	0.22	>.99
Control vs. Back-handling	$-$0.64	.97
Sucrose vs. Back-handling	0.49	.99
Tickling vs. Back-handling	$-$0.94	.88
Sucrose vs. Control	1.1	.80
Tickling vs. Control	$-$0.31	>.99
Tickling vs. Sucrose	$-$1.4	.61

The treatment did, however, have an effect on the number of trials initiated (LRT = 9.8, *p* = .04). Post-hoc analyses suggested that finding was driven by a reduced number of trials initiated by rats following sucrose consumption, and increased number of trials initiated by rats following tickling (Tables 2 and 4). The number of trials completed increased significantly as rats completed more test sessions ($\beta \pm$*SE* = 3.495 ± 0.599, LRT= 32, *p*$<.001$).

**Table 4:** Results of the pairwise comparisons of the number of trials completed

Pairwise comparison	*z*-value	*p*-value
Back-handling vs. Air-puff	$-$0.37	>.99
Control vs. Air-puff	$-$0.44	.99
Sucrose vs. Air-puff	$-$2.1	.24
Tickling vs. Air-puff	0.76	.94
Control vs. Back-handling	$-$0.077	>.99
Sucrose vs. Back-handling	$-$1.7	.44
Tickling vs. Back-handling	1.1	.79
Sucrose vs. Control	$-$1.6	.49
Tickling vs. Control	1.2	.75
Tickling vs. Sucrose	2.8	.04

## Discussion

Foraging tasks might provide insight into affective state in non-human animals. Here, we assessed whether rat foraging behavior was influenced by manipulations designed to alter affective state. To achieve this, rats completed a task in which they had repeatedly to decide whether to continue to harvest a food source with increasing time costs, or to forgo food to switch to a different food source. Affect was manipulated in the first experiment by either removing enrichment or implementing unpredictable housing conditions (a version of a chronic mild stress protocol) and then reversing these treatments; these have been found to influence a variety of outcome measures putatively related to affect and are widely used as affect manipulations (Neville, Lind et al., 2023; Willner et al., 1992; Willner, 2017; Hill et al., 2012; Paolo et al., 1994). Then, affect was manipulated in the second experiment by delivering rewards (sucrose or tickling) or punishers (air-puff or back-handling) immediately prior to testing. These manipulations were selected as rewards and punishers are key determinants of affective state according to operational definitions and theoretical conceptualisations of affect.

Training rats to complete this task was relatively straightforward, with all rats completing seven training sessions before progressing to testing. This compares favorably to other decision-making tasks designed to measure affect, such as the judgement bias task, where training takes upwards of 15 test sessions and subject attrition is common (Jones et al., 2018).

We found that foraging behavior was altered when rats were in poorer housing conditions. In these conditions, rats were more likely to switch to the alternative food source rather than continue to harvest the current food source. This was only significant in the enrichment removal but not the unpredictable housing condition, although there was no week in which the difference between the enrichment removal and unpredictable housing was significant, suggesting that the unpredictable housing treatment may have exerted a similar, yet weaker effect than enrichment removal. Thus, within our study, negative affect (induced by enrichment removal) resulted in less persistence at a food source.

This finding is consistent with some (Lenow et al., 2017; Strauss et al., 2011) but not all (van Dooren et al., 2021; Aberg et al., 2022; Blanco et al., 2013; Harlé et al., 2017) human studies that have examined links between affective state and persistence. It is notable that our results align best with the human study from which our methods were derived: the task by Lenow et al. (2017) is framed as a patch foraging task in which participants decide sequentially whether to ‘harvest’ at a tree displayed on screen which depletes with each successive harvest, or ‘switch’ to a new tree for which there is a travel time. This might suggest that the direction of the relationship between affective state and persistence depends at least partially on the framing and context of the decision-making task.

Rats also completed fewer trials in total when in worse housing conditions, an effect that was observed reliably across both the enrichment removal and unpredictable housing manipulations. The number of completed trials and the decision to ‘harvest’ or ‘switch’ are interconnected: rats can complete more trials by making fewer ‘switch’ decisions, which tend to take longer. This is also evidenced by the strongly significant relationship between these two measures. However, the number of trials a rat can complete will also depend on their locomotion speed when traversing the shuttlebox, and the speed at which they initiate new trials at the same trough. The number of trials completed in the patch foraging task might therefore provide a marker of affect, but further research needs to be conducted to understand precisely what aspects of cognition and/or motor control are being altered by affect to influence the number of trials completed.

The effect of the affect manipulations also appeared to act more quickly on the number of trials measure compared to the decision to ‘harvest’ or ‘switch’. For the decision measure, the effect appeared to peak on the second week in the poorer environment, and then overall returned towards baseline levels. In contrast, for the number of trials measure, the peak appeared to occur in the first and second week in the poorer environment, and returned to (and overshot in absolute terms) baseline levels. It is not completely clear how to make a direct statistical comparison between these time courses; however, it is an obvious target of future studies.

Similarly, while there was no evidence that the short-term affect manipulations influenced foraging decisions (i.e., ‘harvest’ or ‘switch’), there was evidence that they influenced the number of trials completed. Rats who were tickled prior to testing completed more trials than those provided with sucrose prior to testing. The effect of tickling on number of trials is consistent with the effect of the long-term manipulations: relatively more positive affect leads to more trials being completed, and conversely relatively more negative affect leads to fewer trials being completed. While in opposition to this, the effect of sucrose on number of trials completed is hardly surprising — a rat that has eaten sucrose prior to testing may be less motivated to complete trials to obtain sucrose during testing — and does not necessarily reflect a change in affect. This raises an important point, which is that although the number of trials is a one-dimensional output measure, its interpretation in terms of affect may not be straightforward because it is likely influenced by an amalgamation of multiple variables or processes, not all of which may be directly related to affect. Therefore, it is important to remain cautious when interpreting the number of trials completed as a potential indicator of affect.

The effect of the short-term manipulations was however quite weak: none of the pairwise comparisons between control and treatment groups were significant, and the two negative short-term affect treatments appeared to have no or little effect on foraging behavior at all. It is possible that foraging behavior may be more sensitive to chronic as opposed to more transient changes in affect, or that the particular batch of rats used for this study were more resilient to the short-term negative manipulations used (noting that similar manipulations have been found to alter behavior on other decision-making tasks in rats within our laboratory, and that these studies were used to inform the power calculations Neville et al., 2020).

Our study is also subject to the perennial problem of not knowing the ground truth of the affective state experienced by the animals. Although our affect manipulations were selected on the basis that rats overall find them to be rewarding (e.g., approached, or selected in preference tests; Neville, Lind et al., 2020, 2023; LaFollette et al., 2017), punishing (e.g., avoided; Lorenzini et al., 1990; Neville et al., 2020), or that their implementation induces behaviors that parallels depression in humans (e.g., anhedonia; Willner, 2017), we can never be absolutely certain of the valence of the affective state induced. The issue of individual differences further complicates this: even if a manipulation does on average have a positive or negative impact on affect, its impact may differ between individuals. Some individuals may be more resilient to manipulations designed to induce negative states (Rygula et al., 2013), and there is likely individual variation in the extent to which ’tickling’ is perceived as rewarding (LaFollette et al., 2017; Hinchcliffe et al., 2020; Neville, Lind et al., 2023). Likewise, behaviors considered to measure affective state might be strongly influenced by individual differences (Brooks et al., 2024; Richter & Hintze, 2019). For example, immobility in the forced swim test and defensive burying, which have been used as measures of anxiety-like behavior in rodents, depend on the extent to which an individual adopts a proactive or reactive coping style (Koolhaas et al., 2010).

Most critically, rats did not complete the task as we had anticipated. Although rats did visit the two troughs, staying at the same trough for a variable number of consecutive trials before switching to the alternative trough, these decisions were interspersed with periods in which the rat was not engaged with the task. This disengagement is reflected in the inter-trial interval summary statistics which indicate that rats did not always immediately initiate new trials at the same trough nor move swiftly across the shuttlebox, and was confirmed when we referred to the video footage of rats in the operant boxes. As a result of this, we were not able to model the data as planned because the time taken to ‘switch’ bore little reflection of the cost to the animal of doing so (e.g., in terms of effort expended, or opportunity costs). There is evidence from another rodent species (mice) that individuals have periods of disengagement within decision-making tasks (Ashwood et al., 2022). The lack of food restriction in our study may have exacerbated the rats’ proclivity for disengagement. Exploratory and preliminary analyses of data from this task suggest that the extent to which rats disengage on the task might depend on affective state, with greater disengagement during the enrichment removal and unpredictable housing periods (see Supplementary Material). It would be valuable to further investigate disengagement in future studies, using a task that is specifically designed to do so. This is possible: in tasks assessing the psychometrics of brain stimulation reward in rats (Arvanitogiannis & Shizgal, 2008), time spent not ‘working’, in our terms being disengaged, plays a critical role in the quantification of the behavior (Niyogi et al., 2014).

In sum, we found that rats completed fewer trials and were more prone to switching to an alternative trough when housed in standard housing conditions from which previous enrichment had been removed (thought to induce a relatively negative affective state), compared to enriched housing conditions (thought to induce a relatively positive affective state), and conversely completed the greatest number of trials following tickling (thought to induce positive affect) in a second experiment using short-term affect manipulations. However, we also found that other factors (e.g., motivation; engagement with the task) might muddy interpretation of number of trials initiated, with rats also completing fewer trials when they had eaten sucrose prior to testing. Although foraging behavior has promise as a measure of affect, questions remain about what performance in our foraging task specifically reflects. As such, foraging behavior in the context of our operant task does not yet provide a reliable measure of affective state in animals.

## Supplementary Information

Below is the link to the electronic supplementary material.

Supplementary file 1 (csv 1297 KB)

Supplementary file 2 (csv 908 KB)

Supplementary file 3 (pdf 426 KB)

## Additional Information

### Data Availability

Data is provided within the manuscript or supplementary information files.
