# Supplementary material for: You are How You Eat: Foraging Behavior as a Potential Novel Marker of Rat Affective State
Source: Affect Sci. 2024 Jun 26;5(3):232–45. doi: 10.1007/s42761-024-00242-4 (PMC11461729; doi:10.1007/s42761-024-00242-4)
Supplement: Supplementary file 3 — (pdf 426 KB) [file 42761_2024_242_MOESM3_ESM.pdf]

Supplementary Material:

You are how you eat: foraging behaviour as a potential novel marker of rat affective state (submitted to Affective Science)

Vikki Neville[1*], Emily Finnegan[1], Elizabeth S. Paul[1], Molly Davidson[1], Peter Dayan[2],

Michael Mendl[1]

[1] Bristol Veterinary School, University of Bristol, Langford, United Kingdom

[2] Max Planck Institute for Biological Cybernetics & University of Tubingen, Tubingen, Germany

* vikki.neville@bristol.ac.uk

1**A. Photographs of the enriched cages (side and front views)**

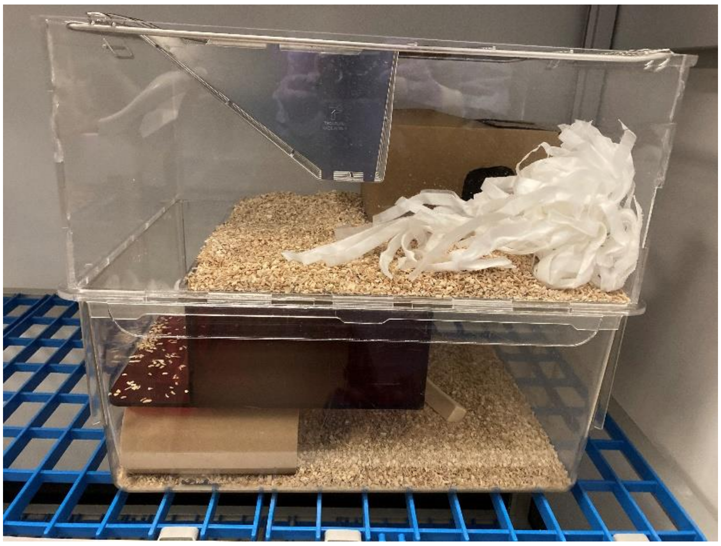

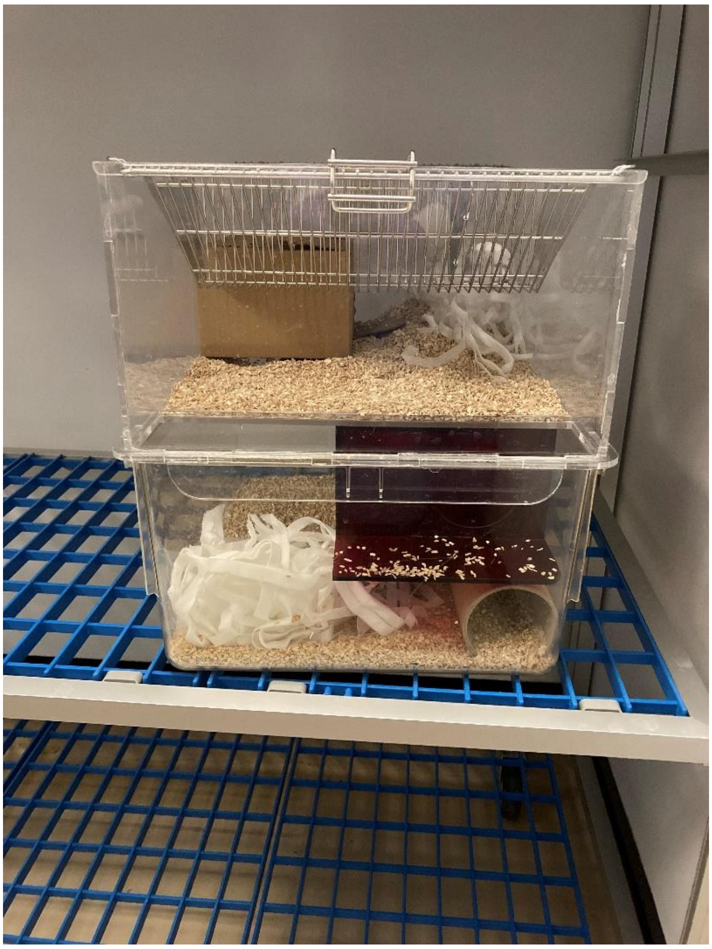

B. Pairwise comparisons between test weeks in Experiment 1

Data	Pairwise comparison	z-value	p-value
Decision: All data	W2 vs W1	2.133	0.333
	W3 vs W1	4.606	<0.001
	W4 vs W1	2.941	0.051
	W5 vs W1	3.113	0.031
	W6 vs W1	2.264	0.262
	W7 vs W1	2.677	0.104
	W3 vs W2	2.438	0.182
	W4 vs W2	0.778	0.987
	W5 vs W2	0.928	0.968
	W6 vs W2	0.099	0.999
	W7 vs W2	0.498	0.999
	W4 vs W3	-1.681	0.629
	W5 vs W3	-1.551	0.713
	W6 vs W3	-2.372	0.210
	W7 vs W3	-1.981	0.427
	W5 vs W4	0.145	0.999
	W6 vs W4	-0.689	0.993
	W7 vs W4	-0.289	0.999
	W6 vs W5	-0.842	0.981
	W7 vs W5	-0.438	0.999
	W7 vs W6	0.405	0.999
Decision: Enrichment only	W2 vs W1	2.669	0.106
	W3 vs W1	5.020	<0.001
	W4 vs W1	3.108	0.031
	W5 vs W1	2.294	0.247
	W6 vs W1	0.419	0.999
	W7 vs W1	3.255	0.019
	W3 vs W2	2.258	0.264
	W4 vs W2	0.360	0.999
	W5 vs W2	-0.478	0.999
	W6 vs W2	-2.276	0.255
	W7 vs W2	0.467	0.999
	W4 vs W3	-1.952	0.445
	W5 vs W3	-2.842	0.067
	W6 vs W3	-4.633	<0.001
	W7 vs W3	-1.875	0.497
	W5 vs W4	-0.868	0.977
	W6 vs W4	-2.710	0.096
	W7 vs W4	0.105	0.999
	W6 vs W5	-1.886	0.490
	W7 vs W5	0.990	0.956
	W7 vs W6	2.862	0.064
Decision: CMS only	W2 vs W1	0.695	0.993

	W3 vs W1	2.105	0.350
	W4 vs W1	1.410	0.797
	W5 vs W1	2.376	0.209
	W6 vs W1	2.653	0.111
	W7 vs W1	0.979	0.959
	W3 vs W2	1.404	0.800
	W4 vs W2	0.710	0.992
	W5 vs W2	1.666	0.639
	W6 vs W2	1.942	0.453
	W7 vs W2	0.277	0.999
	W4 vs W3	-0.697	0.993
	W5 vs W3	0.249	0.999
	W6 vs W3	0.522	0.999
	W7 vs W3	-1.135	0.917
	W5 vs W4	0.953	0.964
	W6 vs W4	1.227	0.884
	W7 vs W4	-0.437	0.999
	W6 vs W5	0.276	0.999
	W7 vs W5	-1.398	0.803
	W7 vs W6	-1.673	0.634
Trial number: All data	W2 vs W1	-3.006	0.042
	W3 vs W1	-3.218	0.022
	W4 vs W1	-1.638	0.658
	W5 vs W1	-0.520	0.999
	W6 vs W1	-0.578	0.997
	W7 vs W1	-0.016	0.999
	W3 vs W2	-0.212	0.999
	W4 vs W2	1.368	0.819
	W5 vs W2	2.486	0.164
	W6 vs W2	2.428	0.187
	W7 vs W2	2.990	0.044
	W4 vs W3	1.580	0.695
	W5 vs W3	2.698	0.099
	W6 vs W3	2.640	0.114
	W7 vs W3	3.202	0.023
	W5 vs W4	1.119	0.923
	W6 vs W4	1.060	0.940
	W7 vs W4	1.622	0.668
	W6 vs W5	-0.058	0.999
	W7 vs W5	0.504	0.999
	W7 vs W6	0.562	0.998
Trial number: Enrichment only	W2 vs W1	-2.405	0.196
	W3 vs W1	-1.867	0.502
	W4 vs W1	-0.581	0.997
	W5 vs W1	0.799	0.985
	W6 vs W1	0.908	0.971

	W7 vs W1	0.552	0.998
	W3 vs W2	0.538	0.998
	W4 vs W2	1.824	0.532
	W5 vs W2	3.204	0.023
	W6 vs W2	3.313	0.016
	W7 vs W2	2.957	0.049
	W4 vs W3	1.286	0.859
	W5 vs W3	2.667	0.107
	W6 vs W3	2.776	0.080
	W7 vs W3	2.419	0.190
	W5 vs W4	1.380	0.812
	W6 vs W4	1.489	0.751
	W7 vs W4	1.133	0.918
	W6 vs W5	0.109	0.999
	W7 vs W5	-0.247	0.999
	W7 vs W6	-0.356	0.999
Trial number: CMS only	W2 vs W1	-1.906	0.476
	W3 vs W1	-2.827	0.070
	W4 vs W1	-1.849	0.514
	W5 vs W1	-1.680	0.630
	W6 vs W1	-1.890	0.487
	W7 vs W1	-0.638	0.996
	W3 vs W2	-0.921	0.969
	W4 vs W2	0.057	0.999
	W5 vs W2	0.226	0.999
	W6 vs W2	0.016	0.999
	W7 vs W2	1.268	0.867
	W4 vs W3	0.977	0.959
	W5 vs W3	1.147	0.914
	W6 vs W3	0.937	0.967
	W7 vs W3	2.189	0.301
	W5 vs W4	0.170	0.999
	W6 vs W4	-0.040	0.999
	W7 vs W4	1.211	0.890
	W6 vs W5	-0.210	0.999
	W7 vs W5	1.042	0.944
	W7 vs W6	1.252	0.874

C. Preliminary and exploratory analyses of disengagement

Please note: as the task was not designed to look specifically at disengagement, these analyses have several limitations, and are for the purpose of demonstration only. Confirmatory analyses of data from a task better designed to study disengagement are needed before any conclusions can be drawn about the link between affective state and disengagement.

Briefly, sequences of inter-trial intervals following the harvest decisions only were classified using a two-state Gaussian hidden Markov model using the SSM module in Python (Linderman, 2019: https://github.com/lindermanlab/ssm). We considered that the state characterised by longer inter-trial intervals reflected trials on which the rat was 'disengaged', and the other state reflected trials on which the rat was 'engaged'. There are accordingly four categories of state transitions, the probability of which is modelled by the hidden Markov model: disengaged to disengaged (e.g., longer inter-trial interval followed by longer inter-trial interval), disengaged to engaged (e.g., longer inter-trial interval followed by shorter inter-trial interval), engaged to disengaged (e.g., shorter inter-trial interval followed by longer inter-trial interval), and engaged to engaged (e.g., shorter inter-trial interval followed by longer inter-trial interval).

As shown in Figure 1, overall, the proportion of engaged to disengaged state transitions increased when rats were in the poorer environment (LRT=9.760, p=0.002). The flipside of this is also true: the proportion of engaged to engaged state transitions decreased (LRT=9.828, p=0.002). However, there was no significant difference in the proportion of disengaged to engaged state transitions (LRT=0.072, p=0.787) or disengaged to engaged state transitions (LRT=0.058, p=0.809).

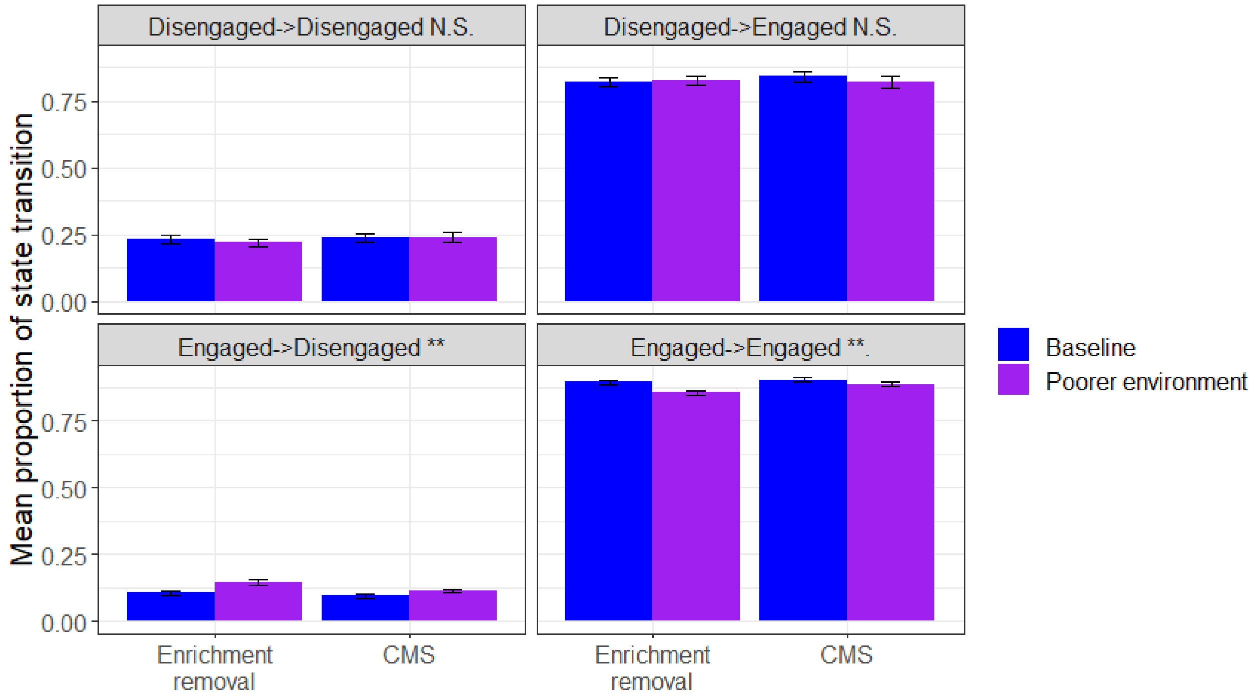

Figure 1. Mean proportion of each type of state transition: 'disengaged' to 'disengaged' (top left), 'disengaged' to 'engaged' (top right), 'engaged' to 'disengaged' (bottom left), or 'engaged' to 'engaged' (bottom right), in the baseline and poorer environment test sessions across the enrichment removal and CMS treatment groups.
